# AMP-Activated Protein Kinase Signalling in Cancer and Cardiac Hypertrophy

**DOI:** 10.4172/2329-6607.1000154

**Published:** 2015-08-31

**Authors:** Yulia Lipovka, John P Konhilas

**Affiliations:** Department of Physiology, University of Arizona, Sarver Molecular Cardiovascular Research Program, USA

**Keywords:** AMP-activated protein kinase, Hypertrophy, Cancer, Cardiotoxicity, Metabolism

## Abstract

The AMP-protein kinase (AMPK) pathway is very versatile as it regulates cellular energetic homeostasis in many different tissue types. An appreciation for the importance of AMPK signalling and regulation in cardiovascular and tumor biology is increasing. Recently, a link has been established between anti-cancer therapy and susceptibility to cardiac disease. It has been shown that some anti-cancer drugs lead to an increased risk of cardiac disease, underlined by de-regulation of AMPK signalling. This review explores the AMPK signalling axis in both cardiac and tumor metabolism. We then examine off-target AMPK inhibition by cancer drugs and how this may translate into increased risk of cardiovascular disease. Finally, we discuss the implication of deregulated AMPK signalling during different stages of cardiac hypertrophy. Better understanding of the molecular pathways behind pathological processes will lead to the development of more effective therapeutics for cancer and cardiovascular diseases.

## Molecular Structure of AMPK

AMP-activated protein kinase (AMPK) is a heterotrimeric complex composed of a catalytic α subunit and regulatory β and γ subunits. Each subunit has at least two different isoforms, which are encoded by distinct genes. They differ slightly in their structure and have differential expression patterns across tissues. The α subunit exists as 2 isoforms (α1 and α2), contains the AMPK serine/threonine kinase domain, and is phosphorylated on at least three residues [1–3]. Phosphorylation of threonine 172 by upstream kinases is essential for AMPK activity, and is often used as an indicator of the activation state of the kinase [4]. Other phosphorylation sites are Thr258 and Ser485, but their contribution to AMPK activity remains to be elucidated [5].The α subunit also has an auto inhibitory domain (AID). The AID interacts with the kinase domain and together they undergo a conformational change in response to AMP interaction with the γ subunit, contributing to AMPK activation [6–8]. AMPKα1 only shares 77% sequence identity with the AMPKα2 isoform [9]. AMPKα1 is primarily found in secreting cells, while AMPKα2 is mainly expressed in skeletal and heart muscle [10].

The β–subunit of AMPK bridges α- and γ-subunits by means of its C-terminal sequence. Its function is not limited to holding the AMPK heterotrimer together, since it contains a central non-catalytic glycogen-binding domain, which senses the status of cellular energy reserved in the form of glycogen [11]. Binding of glycogen with a single glucose α1–6 branch to the β subunit of AMPK allosterically inhibits phosphorylation of α subunit by upstream kinases [12].

AMPKβ-subunit has two isoforms, β1 and β2, that only differ in the first 65 of 275 residues [13]. Despite high structural similarity, they have differential tissue distribution; with β1 being expressed in a wide range of tissues and β2 primarily localized to brain, kidney and striated muscle [14].

The γ subunit can be found as 3 isoforms (γ1, γ2 and γ3) and is made out of four cystathionine β-synthetase (CBS) motifs that pack together generating two Bateman domains (CBS1 + 2 and CBS3 + 4). The symmetry of the CBS domains creates four potential adenyl-binding sites [15]. The 2’ and 3’ hydroxyl groups of each AMP ribose groups interact with an aspartic acid residue located on the first turn of the α-helix adjacent to the site. In the fourth potential adenyl-binding site, an arginine residue is substituted instead, which probably makes AMP binding to this domain impossible. So, mammalian AMPK binds three AMP molecules; one binds to “site 4” and does not exchange for ATP and co-purifies with the proteins since it is tightly bound. The other two AMP molecules compete for binding with Mg-ATP and/ or ATP to sites “1” and “3” and are responsible for adenyl-sensing properties of the mammalian enzyme [16].

**Figure 1:**
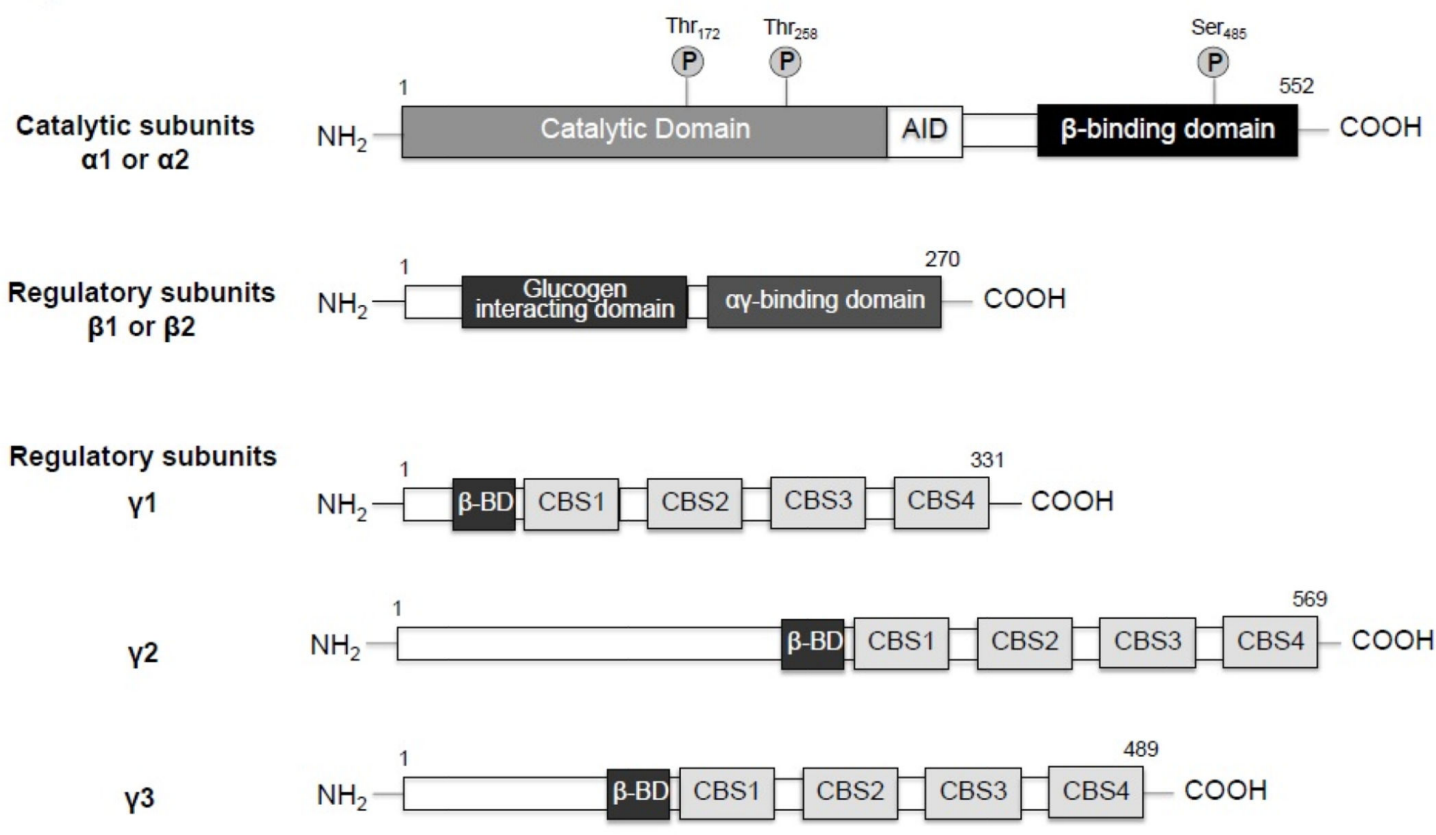
Structure of AMP-activated protein kinase (AMPK) Domain composition of the catalytic (α) and regulatory (β,γ) subunits of AMPK. Phosphorylation sites are shown on the α-catalytic subunit. AID: Auto Inhibitory Domain, CBS: Cystathionine β-Synthetase Motif, β-BD: β-subunit Binding Domain. This representation does not accurately reflect the relative lengths of the subunits and their domains.

The γ isoforms have the greatest structural variability among all AMPK subunits. The most widely expressed isoform is γ1, composed of 331 residues [17]. The γ2 subunit is 569 -residues long and is mainly expressed in the heart, brain, placenta and skeletal muscle [3]. The third isoform γ3, is composed of 489 residues and is only expressed in skeletal muscle [17]. A schematic representation of the three AMPK subunits is presented in Figure 1. Detailed examinations regarding the quaternary structure of AMPK can be found in several studies [9,16,18].

## Mechanisms of AMPK Activation

AMPK activity is regulated in response to the cellular energy state, which is reflected in the ratio of AMP to ATP. During energy usage, ATP is broken down to generate ADP, which can be converted to AMP through the action of adenylate kinase. Binding of AMP facilitates phosphorylation of the activation loop at Thr172 by AMPK kinase (AMPKK) and reduces the dephosphorylation rate of AMPK by the PP2C-a phosphatase [19]. AMP binding to AMPK induces allosteric and conformational changes that affect the interaction between the kinase and the autoinhibitory domains of AMPKα [7,20]. AMP, as the primary activator of AMPK, has a much greater affinity to AMPK than that of ATP even when the cellular concentrations of ATP are much greater than those of AMP. In addition to AMP, ADP can also bind to AMPK, protecting the enzyme from dephosphorylation [10].

**Figure 2:**
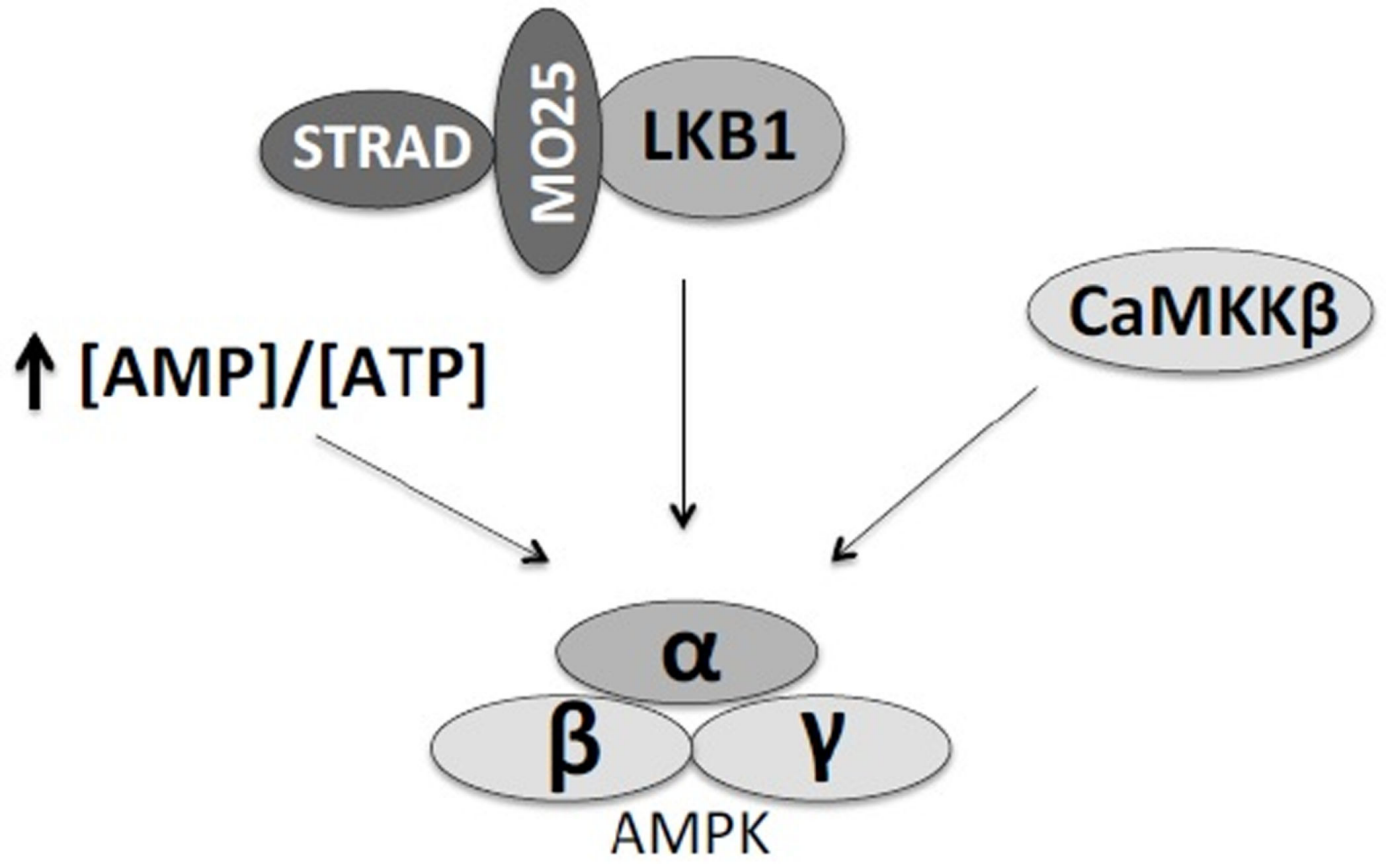
Mechanisms of AMPK activation AMPK is activated in response to an increase in intracellular AMP/ATP ratio, as well as after phosphorylation of its α-catalytic subunit by upstream kinases. The two kinases that phosphorylate AMPK are LKB1, which forms a complex with two accessory proteins STRAD and MO25, and CaMKKβ.

The phosphorylation state of Thr172 reflects the activation status of AMPK and is influenced by the balance between the action of upstream kinases and protein phosphatases. So far, two AMPKKs have been identified: Calcium-calmodulin dependent protein kinase kinase β (CaMKKβ) [21] and the tumor suppressor kinase complex LKB1 [22,23]. The LKB1 complex consists of LKB1 and two accessory subunits, STRAD and MO25, both of which are required for LKB1 activity (Figure 2) [22–24]. There are at least two protein phosphatases that can inhibit AMPK activation: protein phosphatase 2A (PP2A) and protein phosphatase 2C (PP2C). PP2A inhibits AMPK phosphorylation in response to increase in intracellular calcium concentrations [25]. It is not clear what drives PP2C action on AMPK, but alterations in PP2C expression modulate AMPK activation in the heart [26].

In addition to phosphorylation, AMPK can be post-translationally modified by acetylation on its α subunit. Acetylation state of AMPKα is determined by opposing catalytic activities of HDAC1 and p300. Deacetylation enhances the catalytic activity of AMPK by promoting its association with the upstream kinase LKB1 [27]. Post-translational modifications also occur on the regulatory subunits of AMPK. The β subunit can be modified by N-terminal myristoylation of the Gly2 residue. It has recently been proposed that AMP dependent phosphorylation of Thr172 depends on the β subunit of AMPK being myristoylated [28].

## AMPK is Central to Healthy and Pathological Metabolism

### AMPK in cardiac metabolism

**Figure 3:**
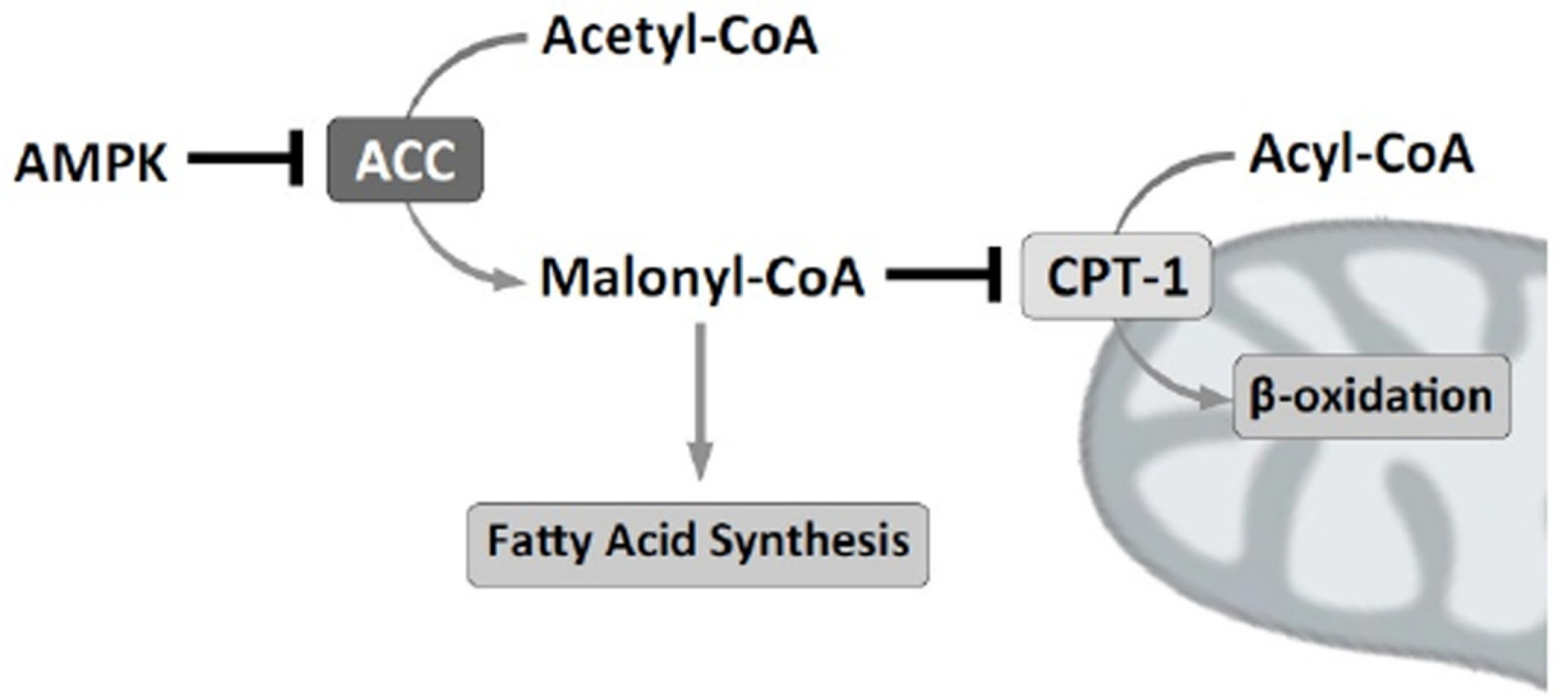
AMPK regulates fatty acid synthesis and β-oxidation AMPK is a negative regulator of Acetyl-CoA carboxylase (ACC) activity. Active ACC catalyzes the conversion of Acetyl-CoA in Malonyl-CoA. Malonyl-CoA allosterically inhibits carnitine palmitoyltransferase-1 (CPT-1), which catalyzes the transfer of the fatty acyl group from acyl-CoA to carnitine, preparing it for transport from the cytosol into mitochondria, where is used for β-oxidation.

Fatty acids are the preferred substrate for energy production in the heart [29]. AMPK modulates cardiac fatty acid metabolism in several ways. As part of this regulatory pathway, AMPK targets and phosphorylates acetyl-CoA carboxylase activity (ACC) inhibiting ACC activity [30,31]. Because ACC catalyses the carboxylation of acetyl-CoA to produce malonyl-CoA, which is a substrate for the biosynthesis of fatty acids, inhibition of ACC activity decreases fatty acid biosynthesis. A major point of regulation of fatty acid oxidation lies in the ability to transport the long-chain fatty acyl-CoA from the cytosol into the mitochondria where it is oxidized to form acetyl-CoA. The rate-limiting enzyme in this process is carnitine palmitoyltransferase (CPT-1). CPT-1 catalyses the transfer of the fatty acyl group from acyl-CoA to carnitine, preparing it for transport from the cytosol into mitochondria. Malonyl-CoA allosterically inhibits CPT-1 activity, impairing the β-oxidation of fatty acids [32]. In summary, ACC inhibition by AMPK decreases malonyl-CoA levels promoting fatty acid transport into mitochondria and increases β-oxidation rates (Figure 3).

AMPK also controls fatty acid transport across the cell membrane in cardiomyocytes. AMPK activation stimulates the expression of fatty acid binding protein (FABPpm) [33]. It also elevates the expression and translocation of the fatty acid transporter FAT/CD36 from intracellular stores to the plasma membrane [34]. Lastly, AMPK stimulates mitochondrial biogenesis, by yet not fully understood mechanisms [35].

**Figure 4:**
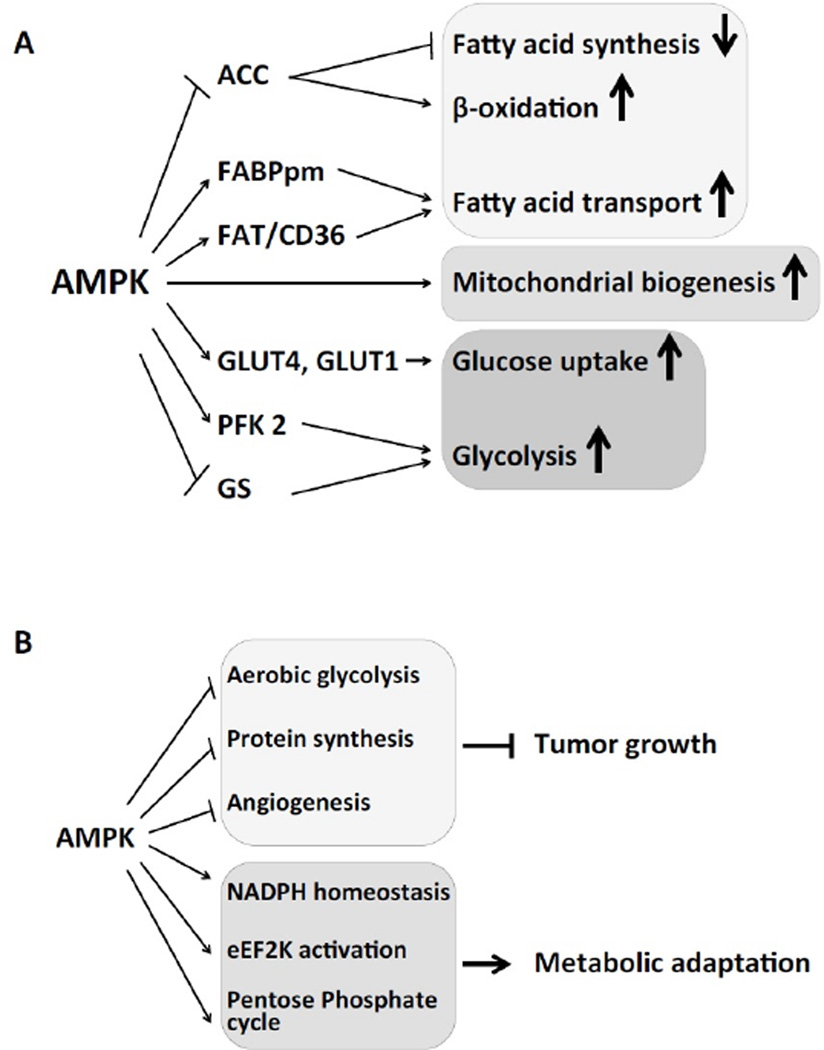
AMPK regulates cardiac and tumor metabolism (A) A schematic representation of the metabolic processes in the heart affected by AMPK and the molecular targets that mediate those effects. ACC: Acetyl-CoA Carboxylase, FABPpm: Plasma Membrane Fatty Acid-Binding Protein, FAT: Fatty Acid Translocase, GLUT4: Glucose Transporter type 4, GLUT1: Glucose Transporter type 1, PFK 2: Phosphofructokinase 2, GS: Glycogen Synthase. (B) A summary of the metabolic pathways affected by AMPK in tumor cells. AMPK can inhibit tumor growth by blocking the metabolic switch to aerobic glycolysis, blocking protein synthesis and reducing angiogenesis. On the other side, AMPK can promote metabolic adaptation of cancer cells by regulating NADPH homeostasis, activating eEF2K and stimulating non-oxidative pentose phosphate cycle.

Another pool of ATP production in the heart is generated by glucose metabolism. AMPK increases glucose uptake by enhancing glucose transporter 4 (GLUT4) and GLUT1-mediated transport [36–38]. AMPK can also phosphorylate 6-phosphofructo-2-kinase (PFK 2), an enzyme responsible for regulation of glycolysis and gluconeogenesis. The result is a net increase in glycolysis during states of energetic stress such as occurs during myocardial ischemia or exercise [39,40]. AMPK also influences glucose storage, by phosphorylating and inactivating glycogen synthase (GS), thus promoting glucose flux through glycolysis [41,42]. A summary of the metabolic pathways affected by AMPK in the heart is presented in Figure 4A.

### AMPK in tumor metabolism

The link of AMPK signalling to cancer dates back to the discovery of LKB1. LKB1 was first identified as a tumor suppressor mutated in an inherited cancer susceptibility known as Peutz-Jegher’s syndrome [43,44]. More recently, it has also been linked with certain types of breast cancer [45]. It is not surprising that AMPK signalling is implicated in cancer metabolism considering that tumor cells must adjust their metabolism to generate the energetic and biosynthetic intermediates required to support increased cell division in the context of stress, such as hypoxia and nutrient deprivation [46]. Fundamental changes in cancer metabolism include a switch to aerobic glycolysis, known as the Warburg effect [47] and increased use of glutamine for mitochondrial-dependent ATP production [48]. AMPK and LKB1 are both negative regulators of aerobic glycolysis. Loss of LKB1 or AMPK activity promotes enhanced glucose and glutamine metabolism, boosting growth and biosynthetic capacity of tumor cells, by increasing HIF-1α expression [49,50].

Activation of the LKB1/AMPK pathway can sometimes give the cells the selective advantage to proliferate, and explains why in some cancers, increased AMPK activity is associated with poor prognosis [51,52]. AMPK can promote metabolic adaptation that supports tumor growth. During energy stress, the generation of NADPH by the pentose phosphate pathway is impaired. AMPK activation at low intracellular ATP levels induces alternative routes for NADPH generation. This is achieved by inhibiting ACC and therefore maintaining NADPH levels by decreasing its consumption in fatty-acid synthesis and increasing its generation by means of fatty acid oxidation [53,54]. AMPK can also activate the eukaryotic elongation factor 2 kinase (eEF2K), which confers cell survival under acute nutrient depletion by blocking translation elongation [55]. In aggressive experimental breast cancer tumors, AMPK activation supports tumor glucose metabolism through positive regulation of glycolysis and the non-oxidative pentose phosphate cycle [56]. The dual role of AMPK signalling in cancer cell metabolism is presented in Figure 4B.

### AMPK is a Tumor Suppressor

Reduced AMPK activation is associated with worsening overall prognosis in many cancers and is sometimes linked to increased metastasis [57–59]. An outcome of reduced AMPK signalling is increased cell proliferation irrespective of the molecular energy levels. This is achieved through uncontrolled activation of the mTOR pathway. Under normal conditions, AMPK inhibits mTORC1 signalling by direct phosphorylation of TSC2 [60] and the mTORC1 regulatory subunit, Raptor [61]. LKB1/AMPK dependent inhibition of the mTOR pathway acts as a tumor suppressor in transformed cells, contributing to cell growth inhibition and repression of oncogenic mRNA translation in response to energy stress [62,63]. AMPK tumor suppressor potential also acts through the Akt/FOXO3 signalling axis. Activated AMPK reduces Akt mediated phosphorylation of FOXO3a, activating this transcription factor and leading to inhibition of tumor growth. Reduction of Akt activity also prevents the epithelial-mesenchymal transition of cancer cells, thereby preventing invasion of basement membranes leading to metastasis [64,65].

The tumor suppressor gene p53 is mutated in many cancers and loss of its function is associated with bad prognosis. Recently, a link between AMPKα2 subunit isoform expression and p53 activation has been established. AMPK α2 levels are suppressed in several tumors, including breast cancer when compared to their healthy counterparts [66]. When AMPKα2 expression is restored in those cells, it promotes p53 acetylation via inhibiting the deacetylase activity of SIRT1. This increases p53 stability and induces apoptosis in tumor cells [67].

### Off-Target Inhibition of AMPK by Cancer Drugs Increases the Risk of Cardiac Disease

Cardiotoxicity is one of the adverse effects of cancer treatment. The most common form of cardiotoxicity is cardiomyopathy associated with the use of anthracyclines as chemotherapeutic agents [68]. The mechanisms behind anthracyclines cardiotoxicity are well studied. A widely accepted mechanism of this cardiotoxicity is through formation of reactive oxygen species (ROS) leading to oxidative stress [69]. However, alternative mechanisms of cardiotoxicity have been proposed. One example is deregulation of cardiac AMPK activity. Anthracyclines, such as doxorubicin, cause cardiac damage by accelerating myofilament apoptosis, suppressing myofilament protein synthesis and altering cardiac energy metabolism [70]. The latter is achieved by decreasing phosphocreatine (PCr)/ATP, AMPK expression and activation [71,72].

Cancer “targeted therapies”, including drugs that inhibit tyrosine kinases, are also cardiotoxic. The majority of pharmacological protein kinase inhibitors are competitors for ATP binding. More than 500 protein kinases possess an ATP-binding site [73]. Because of this, many of the kinase targeting drugs are highly non-specific, and can target several different kinases. This lack of target specificity makes many organ systems susceptible to the toxic effects of anti-cancer drugs in addition to the heart.

To date, two receptor tyrosine kinase (RTK) inhibitors (Sunitib and Herceptin) have been reported to negatively impact cardiac AMPK signalling. Sunitib, a drug used to treat renal and gastrointestinal cancer, causes left ventricular dysfunction [74]. More recently it has been shown to induce myocyte injury *in-vivo*, reduce ATP concentration in cardiomyocytes and impair AMPK’s ability to phosphorylate downstream targets in the cell [75]. These findings suggest that off-target inhibition of AMPK accounts, at least in part, for Sunitib cardiotoxicity. Herceptin (trastuzumab), used to treat HER-2 positive breast cancer, impairs cardiac AMPK activation resulting in failure to induce stress-related survival mechanisms [76]. It also lowers intracellular ATP levels in cardiomyocytes, leading to apoptosis, which is further aggravated by TNFα [77].

As mentioned above, some anti-cancer medications that show cardiotoxicity have an inhibitory effect on cardiac AMPK signalling. AMPK is central to the energetic homeostasis of cardiac cells. A decrease in AMPK activation capacity causes a misbalance in energy handling, which could lead to the development of cardiac pathologies, such as hypertrophy. It is therefore important to consider accompanying cancer therapies that would counteract the cardiotoxic effects of anti-cancer agents, with a special focus on balancing cardiac AMPK signalling.

## AMPK Signalling is Implicated in the Initiation and Progression of Cardiac Hypertrophy

Cardiac hypertrophy is a thickening of the heart muscle, which results in a decrease in size of the chamber of the heart, including the left and right ventricles. It is considered an adaptive response of the heart to a number of disease etiologies. The changes in cardiac mass as a result of hypertrophy are associated with changes in cardiac metabolism, which slowly changes its preference for ATP production from fatty acids to carbohydrates, as hypertrophy progresses [78].

Decreased AMPK signalling is associated with an increased risk of developing cardiac hypertrophy. In terms of molecular changes, cardiac hypertrophy is denoted by enhanced protein synthesis, changes in gene transcription and increased myofibrillar assembly [79].

Pharmacological activation of AMPK inhibits protein synthesis and gene transcription associated with cardiac hypertrophy [80,81]. Inactivation of AMPK in neonatal rat cardiomyocytes is permissive to development of hypertrophy [82]. This is denoted by AMPK ability to inhibit mTOR signalling [80]. Similarly, a decrease in AMPK activity exacerbates hypertrophic growth and heart failure following transverse aortic constriction [83].

The role of AMPK activation during the progression of left ventricular hypertrophy (LVH) remains controversial, since it stimulates a response that in some cases may be adaptive, while in others, maladaptive. AMPK is activated in models of chronic pressure overload and linked to a switch in substrate preference to glucose, by an underlying increase of GLUT4 in the plasma membrane [84]. In this model, increased glucose uptake and increased AMPK activity are associated with the development of cardiac hypertrophy [85]. In contrast, in a model of spontaneously hypertensive rats AMPK activation is linked to inhibition of LVH development [86]. The effect on metabolic pathways was not examined in this model. It is very likely, that the cardio-protective effect of AMPK activation in this model is mediated by inhibition of pro-hypertrophic signaling. This is mainly achieved by targeting the mTOR pathway [81,82].

## Concluding Remarks

AMPK signalling sits at the nexus of cellular energy sensing and homeostasis in a variety of cell types. It is particularly important in the heart, a highly energy-consuming organ. Alterations in AMPK signalling can trigger a series of downstream molecular events that alter the way heart responds to external stimuli, particularly those stimuli that lead to energetic stress. Depending on the nature of the energetic stress, long- vs. short-term or pathological vs. physiological, AMPK signalling can either promote or attenuate the development of cardiac disease. External factors, such as anti-cancer drugs. deregulate cardiac AMPK signalling leading to unwanted and potentially harmful cardiovascular side effects. Future studies are needed to fully characterize all anti-tumor agents that affect cardiac AMPK signalling and negatively impact cardiac health. Pharmacological modification of currently available drugs and development of new cancer therapeutics is a key step to more effective treatment regimens.

## Abbreviations

β-BD: Beta-Subunit Binding Domain
ACC: Acetyl-CoA Carboxylase
AID: Auto Inhibitory Domain
Akt: Protein kinase B
AMPK: AMP-Activated Protein Kinase
AMPKK: AMP-Activated Protein Kinase Kinase
CaMKKβ: Calcium-Calmodulin Dependent Protein Kinase Kinase Beta
CBS: Cystathionine β-Synthase
CPT-1: Carnitine Palmitoyl Transferase
eEF2K: Eukaryotic Elongation Factor 2 Kinase
FABPpm: Fatty Acid Binding Protein
FAT/CD36: Fatty Acid Translocase
FOXO3: Forkhead Box O3
GLUT1: Glucose Transporter 1
GLUT4: Glucose Transporter 4
GS: Glycogen Synthase
HER-2: Human Epidermal Growth Factor Receptor 2
HDAC1: Histone Deacetylase 1
HIF-1α: Hypoxia-Inducible Factor 1 Alpha
LKB1: Liver Kinase B1
LVH: Left Ventricular Hypertrophy
MO25: Mouse Protein 25
mTOR: Mammalian Target of Rapamycin
mTORC1: Mammalian Target of Rapamycin Complex 1
PCr: Phosphocreatine
PFK 2: 6-phosphofructo-2-Kinase
PP2A: Protein Phosphatase 2A
PP2C: Protein Phosphatase 2C
p300: Histone Acetyltransferase p300
ROS: Reactive Oxygen Species
RTK: Receptor Tyrosine Kinase
SIRT1: Sirtuin 1
STRAD: Ste-20-Related Adaptor Protein Complex
TNFα: Tumor Necrosis Factor Alpha
TSC2: Tuberculosis Sclerosis 2
